# Vaginal Fibroblast Behavior as a Function of Stiffness Changes in a Polyisocyanide Hydrogel for Prolapse Repair

**DOI:** 10.1021/acsabm.3c00433

**Published:** 2023-08-17

**Authors:** Aksel N. Gudde, Melissa J. J. van Velthoven, Betül Türkel, Paul H. J. Kouwer, Jan-Paul W. R. Roovers, Zeliha Guler

**Affiliations:** † Department of Obstetrics and Gynecology, Amsterdam University Medical Center−location AMC, Meibergdreef 9, 1105 AZ Amsterdam, The Netherlands; ‡ Reproductive Biology Laboratory, Amsterdam Reproduction and Development, Amsterdam University Medical Center−location AMC, Meibergdreef 9, 1105 AZ Amsterdam, The Netherlands; § Department of Urology, Radboud Institute for Molecular Life Sciences, Radboud University Medical Centre, Geert Grooteplein Zuid 28, 6525 GA Nijmegen, The Netherlands; ∥ Institute for Molecules and Materials, Radboud University, Heyendaalseweg 135, 6525 AJ Nijmegen, The Netherlands

**Keywords:** Pelvic organ prolapse, vaginal fibroblasts, stiffness, polyisocyanide, collagen deposition

## Abstract

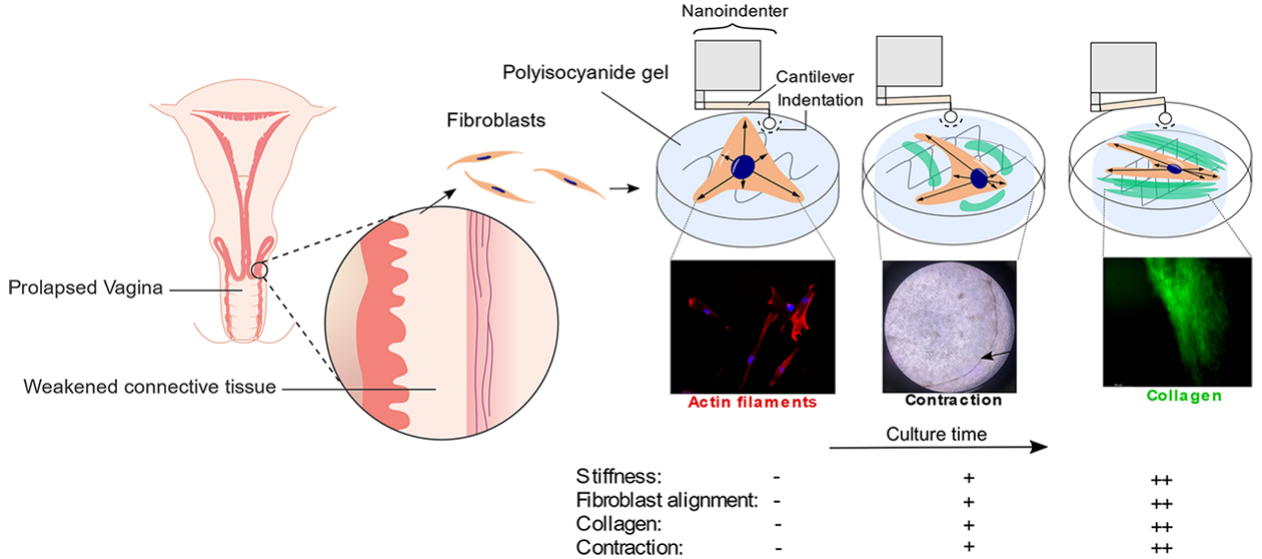

There is an urgent need for improved outcomes in the treatment of pelvic organ prolapse (POP). Success of primary surgery relies on the load bearing capacity of plicated connective tissue underneath the vaginal wall, which is compromised due to an altered vaginal fibroblast function and collagen composition. There is an important factor in connective tissue repair that relates to changes in stiffness of the vaginal fibroblast microenvironment, which influences cell activity through cellular mechanosensing. The aim of this study is to investigate the effect of stiffness changes on vaginal fibroblast functions that relate to connective tissue healing in prolapse repair. The substrate stiffness was controlled by changing the polymer concentration in the fibrous and strongly biomimetic polyisocyanide (PIC) hydrogel. We analyzed stiffness during cell culture and assessed the consequential fibroblast proliferation, morphology, collagen deposition, and contraction. Our results show that increasing stiffness coincides with vaginal fibroblast alignment, promotes collagen deposition, and inhibits PIC gel contraction. These findings suggest that the matrix stiffness directly influences vaginal fibroblast functionality. Moreover, we observed a buildup in stiffness and collagen, with an enhanced fibroblast and collagen organization on the PIC-substrate, which indicate an enhanced structural integrity of the hydrogel-cell construct. An improved tissue structure during healing is relevant in the functional repair of POP. Therefore, this study encourages future research in the use of PIC gels as a supplement in prolapse surgery, whereby the hydrogel stiffness should be considered.

## 1 Introduction

In patients with pelvic organ prolapse (POP), one or more pelvic organs (bladder, uterus and/or bladder) descend into the vagina due to weakening of supportive tissues. POP can be treated by surgery, which is indicated for one out of 10 women during a lifetime.^1^ The primary surgical method is native tissue repair (NTR), which involves the plication of connective tissue underneath the vaginal epithelium to restore the pelvic floor anatomy. However, the load bearing capacity of the pelvic floor is compromised, contributing to a high recurrence rate (32–45%).^2^ The fibroblast function in the vaginal connective tissue of POP patients is altered with impaired production and remodeling of collagen.^3,4^ Studies report that the imbalance in collagen metabolism associates with changes in vaginal stiffness, suggesting an affected mechanical integrity of the tissue.^5−7^

Stiffness is an important parameter in functional healing of vaginal connective tissue, not only as a contributor to being load bearing but also as a property of the vaginal fibroblast microenvironment. Changes in extracellular matrix (ECM) stiffness influence the fibroblast behavior.^8−10^ Earlier work showed that cytoskeletal tension via cell-matrix connections (focal adhesions) affects cell morphology and activates mechanosensitive responses.^11,12^ For fibroblasts, these responses may result in altered collagen deposition that further changes the stiffness of the ECM.^7^ In prolapse repair, such changes in tissue metabolism may result from the insertion of biomaterials with incompatible stiffness properties.^13,14^ Despite the relevance of stiffness as a functional outcome in connective tissue healing and its influence on the cell microenvironment, only a few *in vitro* studies address the effect of stiffness changes on the vaginal fibroblast response.^10,15^ Furthermore, none of these studies involve the use of biomaterial stiffness to stimulate the regenerative behavior of vaginal fibroblasts for prolapse repair.

In our opinion, changing biomaterial stiffness is an effective way to trigger fibroblast activity and collagen metabolism in vaginal connective tissue. In this study, we aim to investigate the vaginal fibroblast function by changing the stiffness of a polyisocyanide (PIC)-hydrogel. A PIC gel is a mechanically biomimetic and thermosensitive material that shows promise for regenerative purposes by its potential to promote vaginal fibroblast activity, steer stem cell differentiation and secretome, and facilitate neovascularization *in vitro*.^16−18^ Recently, we were able to stimulate vaginal fibroblast activity with respect to ECM deposition and remodeling through adjustments in ligand density in the PIC gel.^19,20^ Based on these results, we hypothesize that we can manipulate the vaginal fibroblast functions and improve relevant parameters in prolapse repair by changing the PIC gel stiffness in the cell microenvironment. Understanding the effects of stiffness on fibroblast function may help in initiating such tissue regenerative strategies. We evaluated stiffness of vaginal fibroblast-hydrogel constructs during cell culture and investigated the cell behavior in terms of proliferation, morphology, and collagen deposition. Moreover, as fibroblasts contract the hydrogel, we monitored the hydrogel contraction.^21^

## 2 Materials and Methods

Three PIC hydrogels of different stiffnesses were obtained by increasing the concentration in PIC polymer solutions to 0.2% w/v (PIC-0.2%), 0.4% w/v (PIC-0.4%), and 0.6% w/v (PIC-0.6%).

### 2.1 PIC-Gel Preparations

A PIC polymer decorated with RGD for cell adherence was synthesized using methods as previously described.^21^ Briefly, isocyanide monomers were randomly copolymerized with a 3.3% feed of isocyanides containing azide (N_3_) groups. The viscosity average molecular weight was determined as described previously.^22^ The RGD peptides were conjugated to a DBCO-PEG_4_-NHS linker. Via click chemistry, RGD was conjugated to obtain the PIC-RGD polymer in a concentration of 55 μM (Figure S1). PIC-RGD was sterilized with a 10-min exposure to UV-light and dissolved at a concentration of 6 g L^–1^ (PIC-0.6%) in cell culture medium at 4 °C on a roller for 2 days. PIC-0.6% was further diluted in cell culture medium to obtain 4 g L^–1^ (PIC-0.4%) and 2 g L^–1^ (PIC-0.2%) concentrations. Gelation involved bundle formation of the polymer, which was induced by increasing the temperature beyond 15 °C.^19^

### 2.2 Mechanical Characterization of PIC Gels

#### 2.2.1 Thermo- and Stress-Sensitivity by Rheology

Hydrogel mechanics were characterized using a rheometer (Discovery HR-2, TA Instruments) with a steel parallel plate (diameter = 40 mm, gap = 500 μm). The storage modulus *G*′ as a function of temperature *T* was measured using a temperature ramp from *T* = 5 to 37 °C (heating rate 1 °C min^–1^, frequency ω = 6.28 rad s^–1^, strain γ = 1%). The differential modulus *K*′ was measured under a small oscillatory stress (ω = 62.8–0.628 rad s^–1^), superposed on a much larger prestress (0.4–200 Pa). For each gel, *G*′ was determined at 37 °C.

#### 2.2.2 Effective Young’s Modulus: Nanoindentation

Stiffness differences between the hydrogel surfaces were assessed by nanoindentation (Piuma, Optics11) in terms of the effective Young’s modulus (*E*_eff_) on day 0. PIC-gels were indented in 96-well plates at 37 °C in a noise-canceling box.^20^ Thin piezos with low-stiffness probes (stiffness = 0.027 N m^–1^, radius = 10 μm, Pavone, Optics11) were used and indented the gel surface during a matrix scan of minimally 6 points (100 μm per step). The indentation depth was 10 μm, and the Hertzian model was chosen.^23^

### 2.3 Vaginal Fibroblast Behavior on PIC

#### 2.3.1 Vaginal Fibroblast Seeding

Vaginal fibroblasts were isolated from the anterior vaginal wall of an informed and consented patient undergoing POP surgery as previously described.^24^ 80 μL of liquid PIC-solutions was pipetted in 96-well plates for nanoindentation, contraction, proliferation, and semiquantitative collagen assays. 100 μL of liquid PIC solutions was pipetted in 8-well Nunc lab chambers (Thermo Fisher, USA) for fluorescent morphology and collagen imaging. The culture plates were incubated at 37 °C for gelation. Vaginal fibroblasts were cultured in Dulbecco’s modified Eagle’s medium (DMEM) supplemented with 10% fetal bovine serum (FBS) and 2% penicillin (100 U mL^–1^)/streptomycin (100 mg/mL) (all Gibco-Life Technologies, Thermo Fisher, USA) up to passage 4 and seeded on the hydrogels at a density of 3 × 10^3^ cells cm^–2^. Culture plates containing hydrogels with cells, further referred to as (cell-hydrogel) constructs, were incubated at 37 C and 5% CO_2_ in a humidified environment. Medium was refreshed once every 3 days on a heating plate. The experiments were independently executed three times in triple.

#### 2.3.2 Modulus during Cell Culture

Stiffness of the cell-hydrogel constructs by means of the effective Young’s modulus (*E*_eff_) during culture was determined on days 14, 21, and 28. The construct surfaces were indented following the specifics as described in section 2.2.2.

#### 2.3.3 WST-I Proliferation Assay

Proliferation was assessed on days 1, 7, 14, and 21 using the colorimetric WST-I assay based on the metabolic cell activity that converts tetrazolium salt to formazan. PIC-gels with vaginal fibroblasts were refreshed with 100 μL of a complete medium supplemented with 10% WST-1. After a 90-min incubation at 37 °C, the absorbance of the supernatant was measured using the Synergy H1 microplate reader (Biotek Instruments Inc.) at 605 (reference) and 440 nm (formazan).

#### 2.3.4 Cell Morphology

The cytoskeleton and nuclei of the vaginal fibroblasts were imaged by fluorescent microscopy on days 1, 3, 7, and 21. All steps were performed at 37 °C. The samples were fixed with 4% paraformaldehyde (PFA) for 20 min. The cells were permeabilized with 0.1% Triton X-100. After being washed with PBS, actin filaments were stained with 100 nM Phalloidin Texas Red-X (Sigma-Aldrich, USA) and incubated for 90 min. The samples were washed twice with PBS, and nuclei were stained with 20 nM Hoechst (Thermo Fisher, USA) for 30 min. The samples were imaged with a Leica DM5000B fluorescent microscope (excitation 591 and 350 nm for Phalloidin and Hoechst, respectively). Morphology was further analyzed by means of individual fibroblast circularity (4 × π × area/perimeter^2^) and spreading area on day 1 and the distribution of the actin filament orientation on day 21 (ImageJ version 1.53c, plugin OrientationJ).

#### 2.3.5 Collagen Deposition

Collagen quantification: Collagen production was quantified by colorimetric absorbance readings of collagen bound Picrosirius red (Direct Red 80, Sigma-Aldrich, USA) on days 14, 21, and 28. PIC gels were liquefied by placing the well-plates on ice for 20 min. Liquid PIC solutions from 8 wells containing cells and ECM were pooled in one centrifuge tube. The tubes were centrifuged for 5 min at 2500 g at 4 °C to remove PIC from the precipitated ECM. The ECM was stored overnight in a polyethylene glycol solution at 4 °C. The samples were washed by 5 min of centrifugation at 3000 g and stained with Picrosirius red for 20 min. The stained precipitate was washed with PBS by a 5-min centrifugation at 3000 g and extracted with buffer (Chondrex). Absorbance was measured at 540 nm by the Synergy H1 microplate reader (Biotek Instruments Inc.). Values were subtracted with background (only PIC gel) and normalized to a standard rat collagen-I standard solution (Cultrex, R&D Systems, USA).

##### Collagen Imaging

Fluorescent imaging of deposited collagen was performed on days 14, 21, and 28 using the CNA35-OG488 dye (CNA), which specifically binds to collagens, such as collagen types I, III, and IV.^25^ Fibroblasts on PIC-gels were fixed in 4% PFA. Samples were washed with PBS and stained with CNA, which was diluted in PBS at a ratio of 1:50, and incubated overnight. After being washed three times with PBS, fibroblast nuclei were stained with Hoechst. Images were taken using the Leica DM5000B fluorescent microscope (excitation 488 and 350 nm for collagen and nuclei, respectively).

#### 2.3.6 PIC-Gel Surface Contraction

Hydrogel contraction, due to contracting fibroblasts, was determined on days 14, 21, and 28 from photographs of PIC gels through a microscopy lens at 5× magnification. The contraction was calculated based on the area percentage of the well occupied by hydrogel using ImageJ software as previously described.^26^ The contracted hydrogel region was determined by the perimeter that followed contracting fibroblasts at the hydrogel edge (Figure S2).

### 2.4 Statistical Analysis

Data were presented and analyzed using the Graphpad Prism version (GraphPad Software, La Jolla, CA, USA). If data passed the D’Agostino-Pearson normality test, results were presented by the mean ± the standard deviation (SD). A two way ANOVA was performed with Bonferroni’s posthoc test to compare between time points and PIC-gels. For data that concerned only one time point, one way ANOVA was selected with Bonferroni’s posthoc test. In the case of skewed data, results were presented in median and interquartile range (IQR). Then, a Kruskal–Wallis test with Dunn’s test was used to compare between time points and hydrogel-groups. Differences were considered statistically significant if *p* < 0.05.

## 3 Results

### 3.1 PIC-Hydrogel Mechanics

#### 3.1.1 Temperature and Stress Dependency

**Figure 1:**
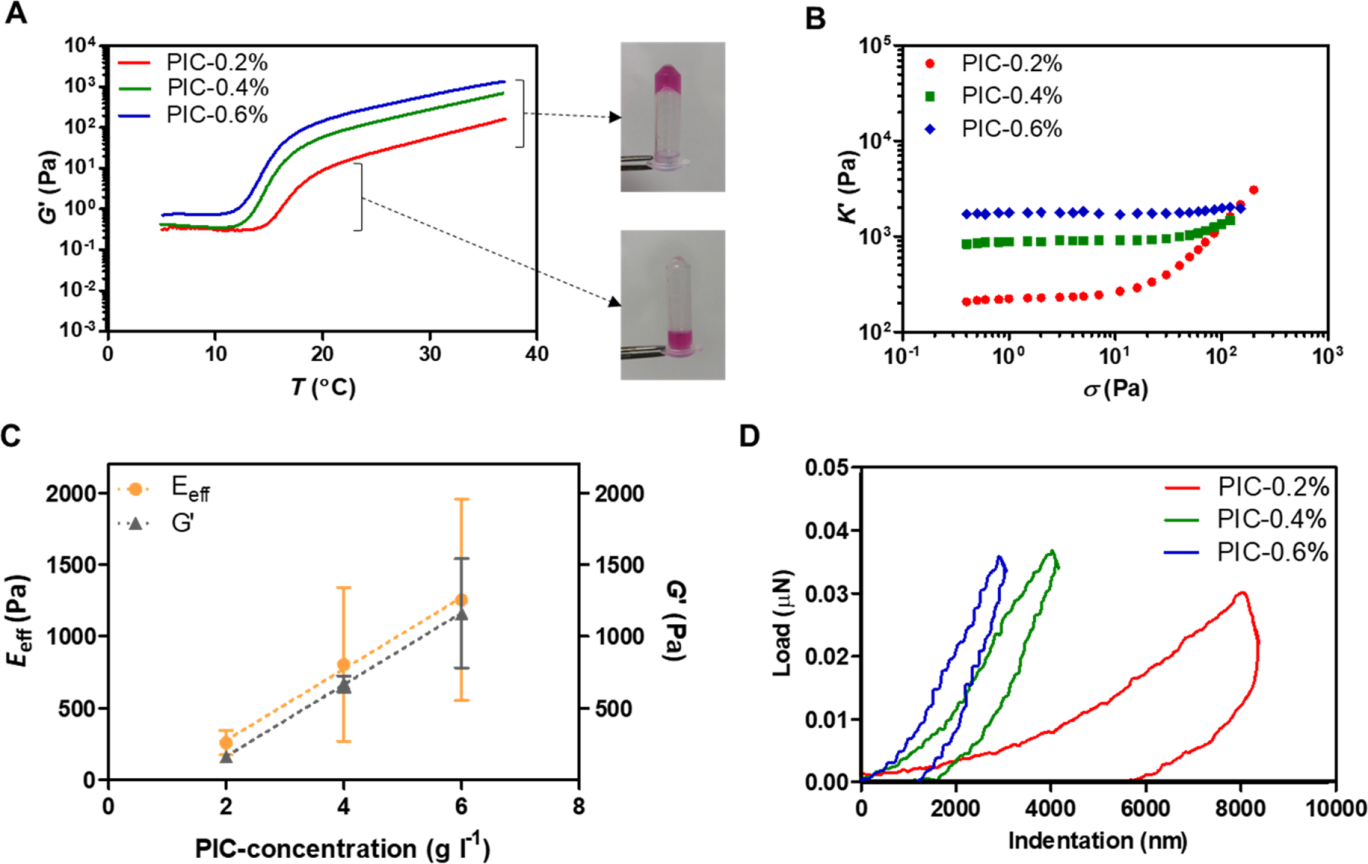
(A) Rheology data of storage shear modulus *G*′ vs temperature *T* and (B) differential modulus *K*′ vs stress σ. (C) Effective Young’s modulus (*E*_eff_) measured by nanoindentation on day 0 and *G*′ as functions of PIC-concentration. (D) Typical load-indentation curves for PIC-0.2%, PIC-0.4% and PIC-0.6%.

The hydrogel characteristics are depicted in Table S1. Figure S1 illustrates the chemical structure of the PIC-RGD polymer. Rheology revealed thermoresponsive and stress-responsive behavior. The thermosensitivity of the gels is shown by the relation between the storage shear modulus *G*′ and temperature (*T*) (Figure 1A). *G*′ of all gels increased strongly above approximately 15 °C, indicating temperature induced gelation.

Under applied shear stress, PIC-gels exhibited a stiffening response that is characterized by an increase in the differential modulus *K′* above a critical stress (Figure 1B). The stiffening was stronger at lower PIC-concentrations, where a critical stress could be quantified for PIC-0.2% and PIC-0.4% (σ_c_; Table S1). With regard to *G*′ at 37 °C, the rheology results display an increasing trend with an increasing polymer concentration (Figure 1C).

#### 3.1.2 Effective Young’s Modulus

Nanoindentations across the hydrogel surfaces on day 0 (37 °C) show an increasing effective Young’s modulus (*E*_eff_) with an increasing polymer concentration (Table S1), confirming differences in substrate stiffness at the start of the cell culture (Figure 1C). Examples of the load-indentation curves display steeper slopes with an increasing polymer concentration (Figure 1D).

#### 3.1.3 Effective Young’s Modulus during Culture

**Figure 2:**
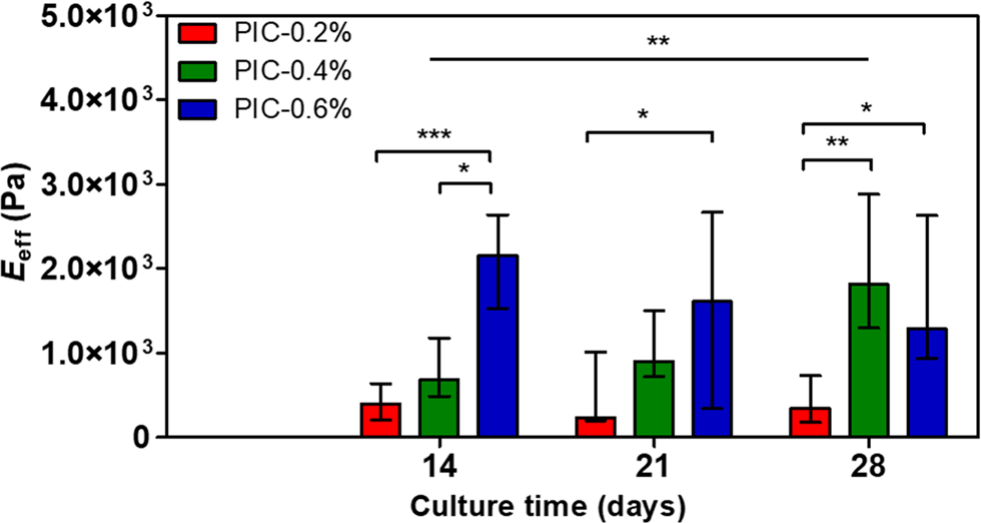
Effective Young’s modulus (*E*_eff_) of PIC-0.2%, PIC-0.4%, and PIC-0.6% substrate surfaces during a 28-day culture with vaginal fibroblasts. *n* = 9 except for day 28 (PIC-0.2% and PIC-0.6%, both *n* = 8). Data is shown in median and IQR. Significance was calculated with the Kruskal–Wallis test, where * *p* < 0.05; ** *p* < 0.01; *** *p* < 0.001.

After seeding PIC gels with the vaginal fibroblasts, assessment of the change in effective Young’s modulus (*E*_eff_) over time revealed an increase for PIC-0.4% (day 14 vs day 28: *p* = 0.003). In contrast, *E*_eff_ values of the other cell-hydrogel constructs were constant in time (Figure 2).

When looking per time point, the surface stiffness increased with increasing PIC concentration on days 14 and 21. Specifically, the PIC-0.6% construct exhibited a significantly increased modulus on these days with respect to PIC-0.2% (days 14 and 21: *p* < 0.001 and *p* < 0.05, respectively) and PIC-0.4% (day 14: *p* < 0.05). However, on day 28, the PIC-0.4% construct modulus increased significantly as compared to PIC-0.2% (*p* < 0.01).

### 3.2 Proliferation and Morphology

#### 3.2.1 Proliferation

**Figure 3:**
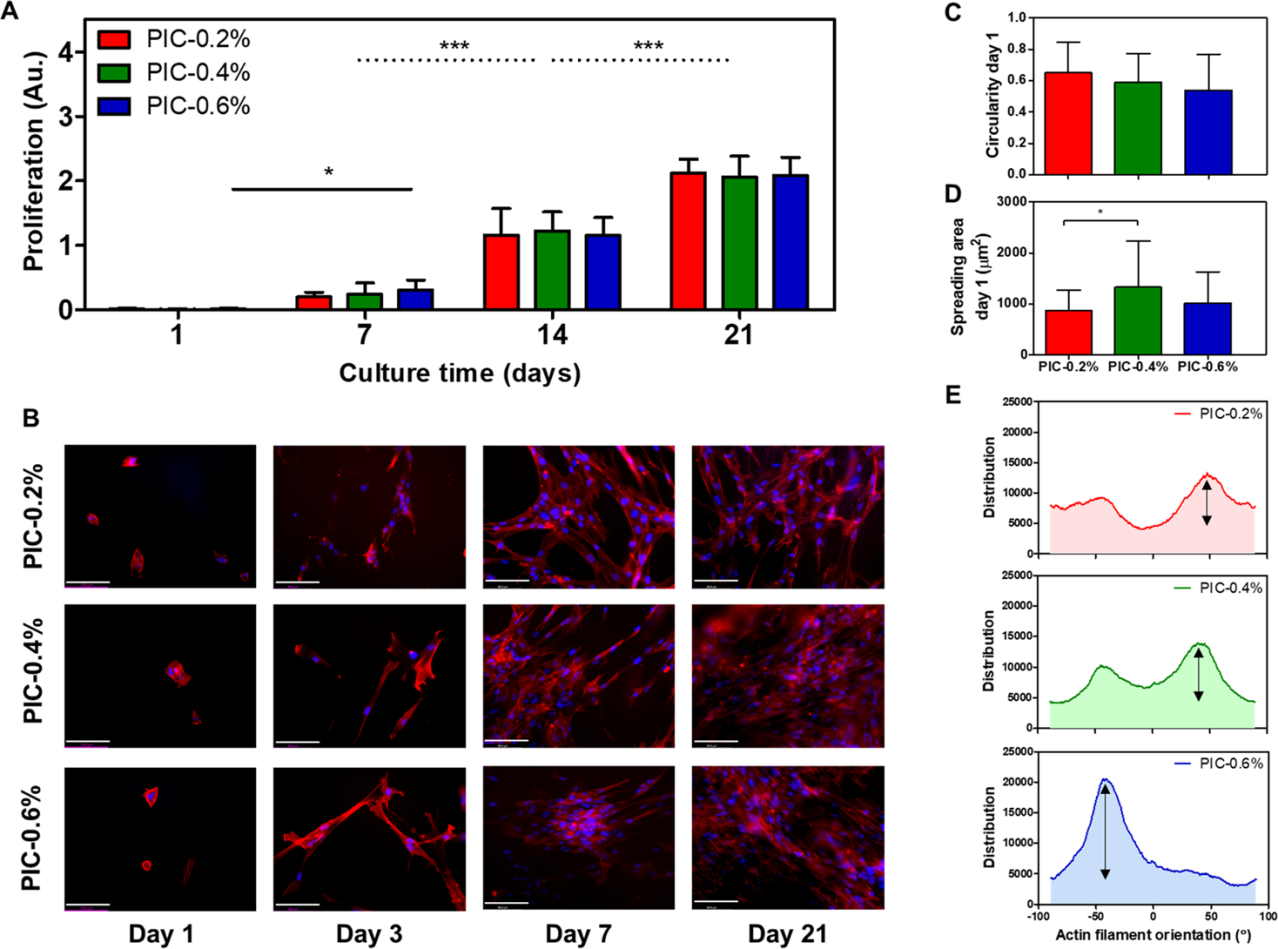
(A) Vaginal fibroblast proliferation during 21 days of cell culture on hydrogels with PIC concentrations in 2, 4, and 6 g L^–1^ (*n* = 9). (B) Fluorescent images of the vaginal fibroblasts on the corresponding hydrogels on days 1, 3, 7 and 21 (scale bars = 100 μm) with actin filaments and nuclei stained by Phalloidin (red) and Hoechst (blue), respectively. (C) Fibroblast circularity between fibroblasts was similar on day 1 (*n* > 25). (D) Cell spreading area on day 1 indicates slight differences between PIC gel stiffness (*n* > 25). (E) Distribution of the actin filament orientations reveals dominant directions characterized by a small peak for PIC-0.2%, two pronounced peaks for PIC-0.4%, and the largest peak for PIC-0.6%. Data is shown in mean ± SD. Significance was calculated with a one way (C and D) and two way (A) ANOVA, where * *p* < 0.05; *** *p* < 0.001.

Vaginal fibroblast proliferation increased on all three hydrogel stiffnesses over time (Figure 3A). However, between days 1 and 7, only fibroblasts on the stiffest gel (PIC-0.6%) proliferated with a significant increase (*p* < 0.05). After day 7, the proliferation increase was significant between time points for fibroblasts on all hydrogels (*p* < 0.001). Comparing between the PIC gels on day 7 revealed that with higher stiffness, fibroblast proliferation tended to increase (PIC-0.2% = 0.20 ± 0.08; PIC-0.4% = 0.24 ± 0.18; PIC-0.6% = 0.31 ± 0.16), albeit insignificantly. The hydrogel stiffness did not affect fibroblast proliferation on the other time points.

#### 3.2.2 Vaginal Fibroblast Morphology

Fluorescent images of actin filaments over time showed that vaginal fibroblasts attached and spread on all gels (Figure 3B). Comparing the stiffness per day, a higher stiffness did not affect cytoskeletal elongation on day 1 (inversely proportional to circularity, Figure 3C). However, the fibroblast spreading area on PIC-0.4% significantly increased as compared to PIC-0.2% (*p* < 0.05, Figure 3D). Furthermore, fibroblasts on the stiffest substrate (PIC-0.6%) tended to align more compared to the other gels (PIC-0.2% and PIC-0.4%) after 7 days. More specifically, fibroblasts on the stiffer PIC-0.6% showed a stronger tendency to orient and align toward a certain direction in contrast to more evenly distributed filament orientations on PIC-0.4% and PIC-0.2% (Figure 3B, Figure S3). Figure 3E illustrates that this trend resulted in one dominant orientation of fibroblast actin filaments for the former substrate on day 21.

### 3.3 Collagen Deposition

#### 3.3.1 Collagen Content

**Figure 4:**
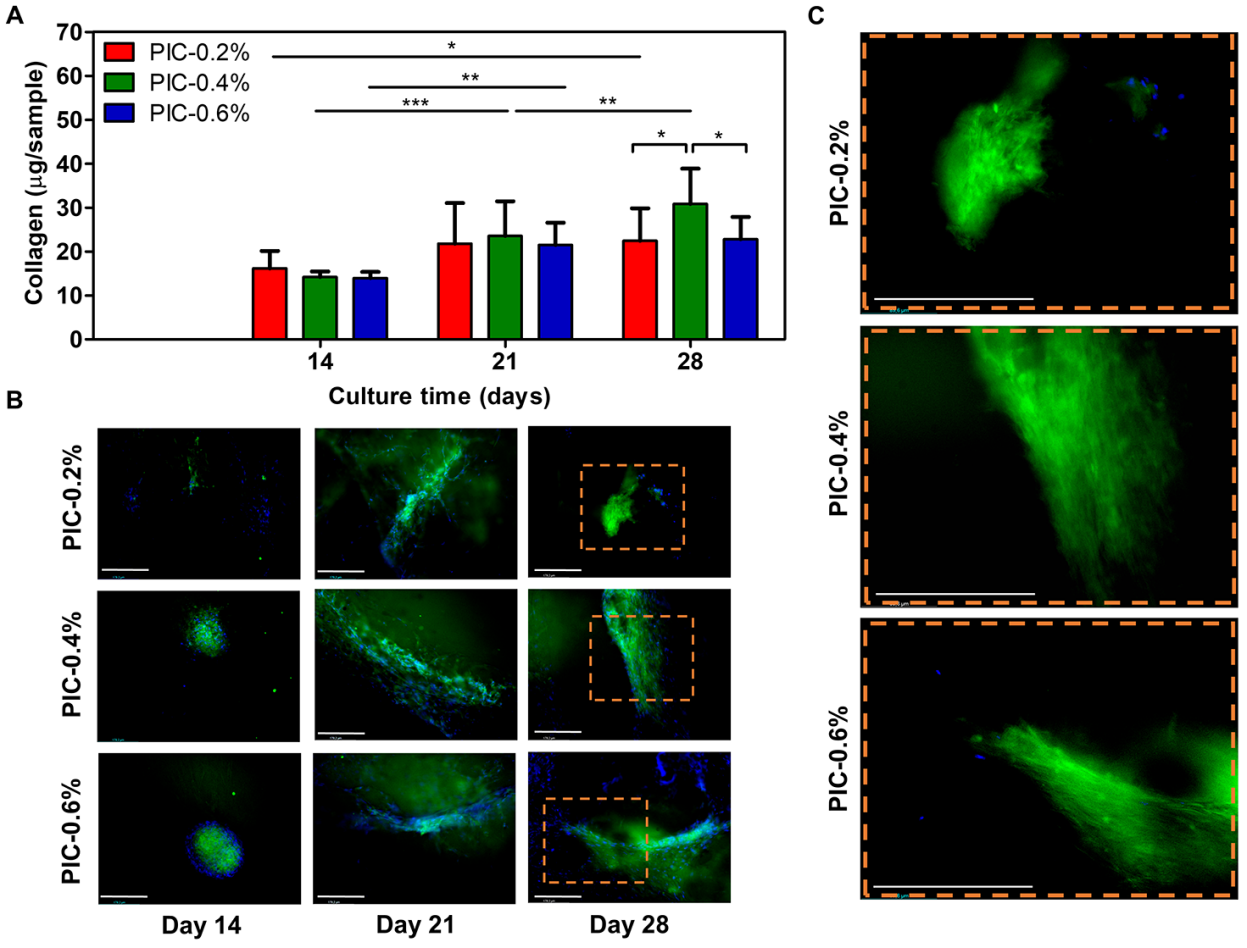
(A) Collagen produced by the vaginal fibroblasts on days 14, 21, and 28 on the PIC-gels (*n* = 9). (B) Fluorescent images of the total collagen structure stained with CNA-OG488 (green) and nuclei (blue) on days 14, 21, and 28 show an increasing collagen production over time. (C) Larger magnification on day 28 displays collagen alignment when PIC gel stiffness increases (all scale bars = 200 μm). Data is shown in mean and SD. Significance was calculated with a two way ANOVA, where * *p* < 0.05; ** *p* < 0.01; *** *p* < 0.001.

Semiquantitative collagen data shows a significant increase over time for all cell-hydrogel constructs (PIC-0.2%: *p* < 0.05; PIC-0.4%: *p* < 0.001; PIC-0.6%: *p* < 0.01; Figure 4A). In addition, vaginal fibroblasts on PIC-0.4% deposited significantly more collagen than fibroblasts on the softer PIC-0.2% and stiffer PIC-0.6% on day 28 (both *p* < 0.05).

#### 3.3.2 Collagen Deposition by Fluorescent Imaging

Fluorescent staining of the collagen with CNA confirms an increasing collagen deposition over time on all substrates (Figure 4B). Specifically, collagen concentrated near aggregated vaginal fibroblasts on day 14. After 21 days, more collagen formed, following an aligned pattern of fibroblast nuclei. In comparison, the stiffer PIC-0.4% and PIC-0.6% constructs demonstrated stronger organization of collagen that seemed to align more than the collagen on PIC-0.2% on days 21 and 28, as shown by the fluorescent images at a larger magnification (Figure 4B).

### 3.4 Stiffness Dependent Hydrogel Contraction

**Figure 5:**
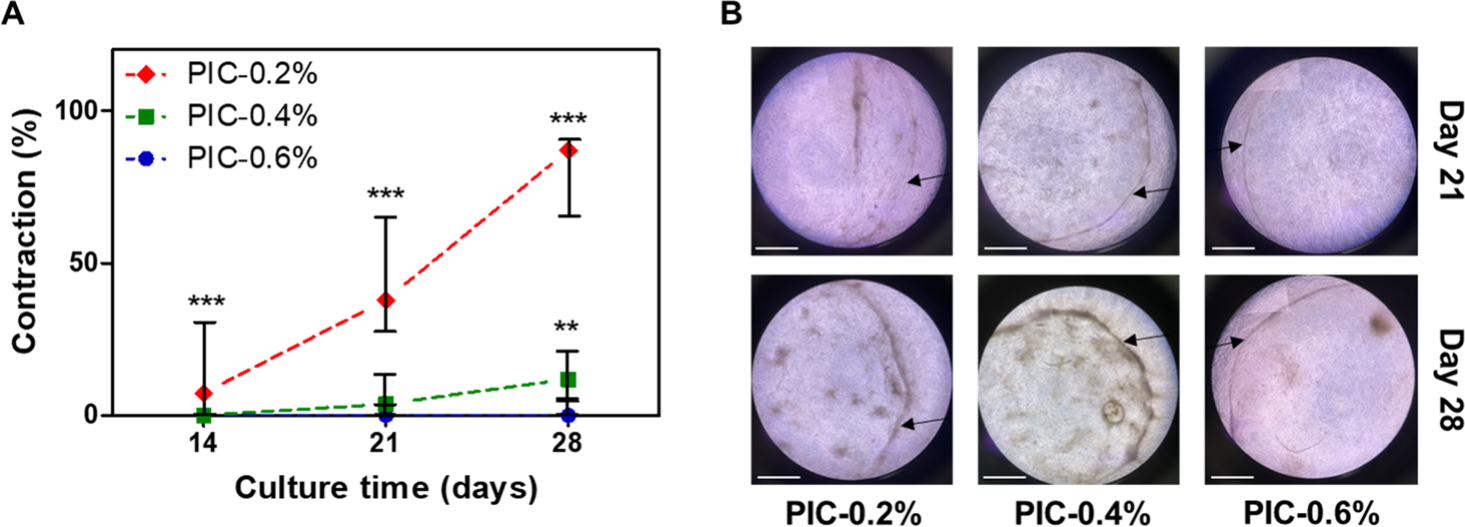
(A) Hydrogel contraction on days 14, 21, and 28 (*n* = 24). (B) Contraction of hydrogel on days 21 and 28 due to contracting vaginal fibroblasts creating a visible edge (black arrows) under light microscopy (scale bars = 500 μm). Data is shown in median and IQR. Significance was calculated with the Kruskal–Wallis test, where ** *p* < 0.01; *** *p* < 0.001.

Contraction was characterized by the appearance of a hydrogel edge with high densities of elongated vaginal fibroblasts (Figure S2). Contraction started for the softest PIC-gel (PIC-0.2%) on day 14 (Figure 5A) and on day 21 for PIC-0.4% and PIC-0.6% (Figure 5B). Furthermore, hydrogel contraction increased significantly over time for PIC-0.2% (day 14 vs 21 and day 21 vs 28: *p* < 0.05) and PIC-0.4% (day 14 vs 21: *p* < 0.001 and day 21 vs 28: *p* < 0.05). Contraction decreased with an increasing initial gel-stiffness, which reached significance between constructs on day 28 (PIC-0.2% vs PIC-0.4% and PIC-0.4% vs PIC-0.6%: *p* < 0.001 and *p* < 0.01, respectively)

## 4 Discussion

In this study, we manipulated the microenvironment of vaginal fibroblasts through alterations in PIC-gel stiffness to influence cell functions. Parameters that relate to a regenerative response *in vitro* changed noticeably. A stiffness increase of the gel-cell construct coincided with alignment and directionality of vaginal fibroblasts, an enhanced collagen deposition, and inhibition of hydrogel-contraction.

The bulk and surface measurements of the selected PIC-gels were correlated with the polymer concentration. The stiffness outcomes were within range of soft tissues and PIC-substrates that facilitate a sustained fibroblast activity, which supports our choice of the selected PIC-concentrations.^20,21,27^ The shear storage and effective moduli were of the same order of magnitude, despite the experimental differences in deformation mode and length scales.^28^ We note that PIC gels, analogous to biological gels such as collagen and fibrin, display unique nonlinear mechanical responses under complex multiaxial deformation (like nanoindentation), which further emphasizes the biomimetic character of the PIC gels.^29,30^ Furthermore, we note that it is challenging to obtain consistent nanoindentation measurements on soft substrates due to increased sensitivity to noise during indentation, as reflected by the presented variations in the modulus. Additionally, during cell culture, the nanoindenter measures across an uneven PIC-gel surface with a heterogeneous distribution of cells and ECM.

Initial differences in substrate stiffness may influence fibroblast functions, such as proliferation, contraction, and ECM synthesis.^31^ During proliferation, these cell activities additionally change the microenvironment, which in turn may affect the cell responses, suggesting the potential involvement of a feedback loop.^32−34^ In this study, an effect of stiffness on vaginal fibroblast behavior was possibly reflected in the cell morphologies, where an initially higher stiffness slightly increased spreading area on day 1. Furthermore, stiffer PIC-gels promoted fibroblast alignment and directionality, which can be explained by traction on fibroblast focal adhesions at an increased substrate stiffness.^35^ The fibroblasts contract in response, which induces alignment along directions of increased tension.^35,36^ The consequential changes in the cytoskeletal configuration may promote proliferation through cell spreading, cell motility, and regulation of mitogenic signaling pathways.^37−39^ This effect might explain the significant proliferation increase on the stiffest PIC-gel on day 7 as compared with day 1.

Nanoindentation data showed that the stiffness increase over time of the gel-cell construct in the initial intermediate stiffness range (to 805 ± 535 Pa; PIC-0.4%, day 0) reached statistical significance on day 28, in contrast to PIC-0.2% and PIC-0.6%. Furthermore, on this day, the former construct exhibited a significantly higher collagen amount compared with the other hydrogels. There could be an effect of stiffening of PIC-0.4% on collagen deposition in line with previous work which demonstrates that an increasing stiffness enhances collagen expression by fibroblasts, which involves mechanically activated signaling pathways.^33,40^ Furthermore, the organization of collagen likely followed the fibroblast arrangement, which seemed to align more when the PIC-gels were stiffer. In addition to the initial hydrogel stiffness, the consequential fibroblast and collagen structure may aid in the stability of the construct and could limit contraction.^41,42^ Indeed, increasing PIC-gel stiffness delayed contraction by the fibroblasts. As stiffness increases, the constructs logically contract at later time points when fibroblast proliferation further progresses, as more work by actin filaments is needed to deform the gel.^21^ In contrast, the softest gel contracted earlier in the presence of little collagen, as lower contraction forces by proliferating fibroblasts were sufficient for deformation of the construct. The enhanced stiffness, collagen deposition, and fibroblast alignment during culture of the intermediate stiffness gel might be attributed to a concurrent action of hydrogel stiffness that applies a mechanical cue to the respective fibroblast functions.^7,34^ Hydrogel contraction may further contribute to this action by promoting stiffness through the deformation of PIC to a denser configuration.^43^ We speculate that the coinciding collagen, contraction, and stiffness increase could be the result of the aforementioned positive feedback; the vaginal fibroblast activities with respect to collagen production and contraction increase stiffness, which further trigger fibroblast responses. Surprisingly, the stiffening could not be confirmed for the softer gel, despite the strong contraction behavior. This is likely a result of the high probe sensitivity to noise and heterogeneous surfaces on low stiffness substrates, which could hinder obtaining significant differences between time points.

The strength of this study lies in the effort to investigate stiffness as a parameter in *in vitro* regeneration for pelvic floor repair. Stiffness influences vaginal fibroblast behavior in the vaginal wall and plays a role in tissue functioning, evidenced by the affected tissue stiffness associated with POP, menopause, and fibrosis.^10,44,45^ Although there is consensus that vaginal fibroblast behavior is critical in pathophysiology and treatment of prolapse, there are few *in vitro* studies that consider stiffness as variable during regeneration in biomaterial development for prolapse repair.^46^ Using a biomimetic injectable gel as a substrate in the context of prolapse repair aids the novelty. With regard to prolapse repair, the current results are encouraging in terms of the potential of PIC-gel to allow for vaginal fibroblast spreading, deposition of collagen, and buildup of stiffness. A controlled ECM deposition accompanied by a stiffness increase in the connective tissue could improve the structural and mechanical integrity of the repair.^7,14,47,48^ PIC-gel in the intermediate stiffness range performed best in this context as the corresponding cell activity resulted in increased collagen production and effective Young’s modulus over time. Furthermore, the increased concentration of PIC, limited contraction, and increasing collagen and modulus indicate enhanced stiffness and structural integrity of the construct. Moreover, *in vivo*, an improved hydrogel integrity aids to surgical applicability as it is beneficial for handling and improves the contact surface with tissue.^49,50^ Therefore, we intend to use PIC-gel of 0.4% w/v *in vivo* to study the potential of PIC-hydrogel as a healing supplement in POP surgery. We acknowledge that *in vitro* models fall short to simulate *in vivo* physiology, implying that the impact of the gel stiffness on the fibroblast response can only further be emphasized *in vivo* due to the presence of immune components and applied mechanical forces. Still, our results imply that stiffness in the vaginal fibroblast microenvironment affects vaginal fibroblast morphology and collagen metabolism. In turn, the vaginal fibroblast activity influences PIC-gel behavior in terms of a stiffness increase and contraction. Therefore, we emphasize that *in vitro* research to biomaterial solutions in prolapse repair should include stiffness as a variable that affects biomaterial performance.

## 5 Conclusions

In this study, we modified the PIC-gel stiffness in the vaginal fibroblast environment, which accompanied an altered cell function. In the intermediate stiffness range, the hydrogel-fibroblast construct stiffened during cell culture and promoted collagen production compared to an initially higher or lower substrate stiffness. Thus, depending on the initial substrate stiffness, PIC-gel might trigger an increased regenerative response of vaginal fibroblasts with a buildup in stiffness. The outcome of this study underlines the need to consider mechanoresponsive cell behavior in selecting PIC-gel compositions and other biomaterials for regenerative purposes, such as prolapse repair.

## Supporting Information Available

Chemical structure of PIC-RGD; hydrogel contraction determination; color survey of actin filament orientation; characteristic properties of PIC-hydrogels (PDF)

## ABBREVIATIONS

POP: pelvic organ prolapse
NTR: native tissue repair
ECM: extracellular matrix
PIC: polyisocyanide
RGD: Gly-Arg-Gly-Asp-Ser
