# Supplementary material for: Vaginal Fibroblast Behavior as a Function of Stiffness Changes in a Polyisocyanide Hydrogel for Prolapse Repair
Source: ACS Appl Bio Mater. 2023 Aug 17;6(9):3759–67. doi: 10.1021/acsabm.3c00433 (PMC10521013; doi:10.1021/acsabm.3c00433)
Supplement: Supplementary file 1 — mt3c00433_si_001.pdf [file mt3c00433_si_001.pdf]

Supplementary information

Vaginal fibroblast behavior as a function of stiffness changes in a polyisocyanide hydrogel for prolapse repair

*Aksel N. Gudde[a, b], Melissa J.J. van Velthoven[c, d], Betül Türkel[a, b], Paul H.J. Kouwer[d], Jan-Paul W.R. Roovers[a, b], Zeliha Guler[a, b] **

[a] Department of Obstetrics and Gynecology, Amsterdam University Medical Center–location AMC, Meibergdreef 9, 1105, AZ, Amsterdam, the Netherlands

[b] Reproductive Biology Laboratory, Amsterdam Reproduction and Development, Amsterdam University Medical Center–location AMC, Meibergdreef 9, 1105, AZ, Amsterdam, the Netherlands

[c] Department of Urology, Radboud Institute for Molecular Life Sciences, Radboud University Medical Centre, Geert Grooteplein Zuid 28, 6525 GA Nijmegen, The Netherlands.

[d] Institute for Molecules and Materials, Radboud University, Heyendaalseweg 135, 6525, AJ, Nijmegen, the Netherlands

* Correspondence to: Z. Guler, Department of Obstetrics and Gynaecology, Amsterdam UMC–location AMC, University of Amsterdam, Meibergdreef 9, 1105AZ Amsterdam, the Netherlands. zeliha.guler@amsterdamumc.nl

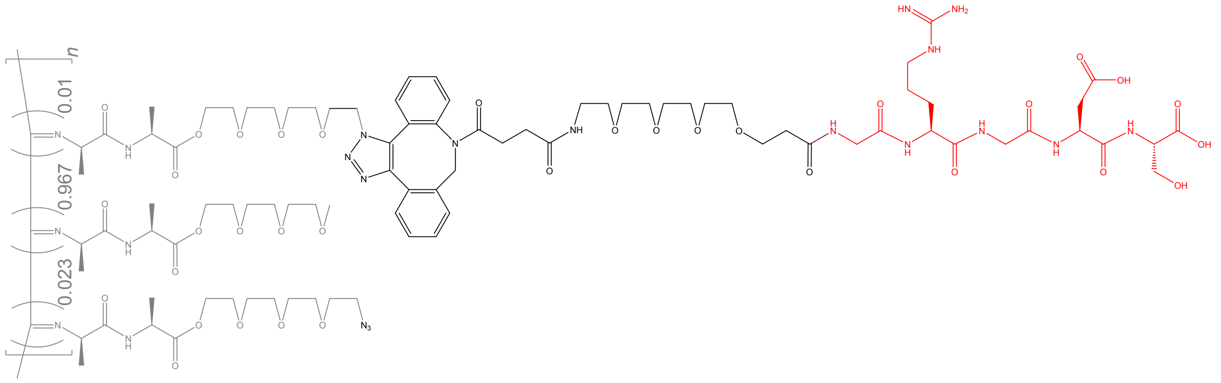

Figure S1. Chemical structure of PIC-RGD with 3.3% feed ratio and 1% RGD conjugation. The black and red part of the structure represent the DBCO-linker and RGD-peptide, respectively.

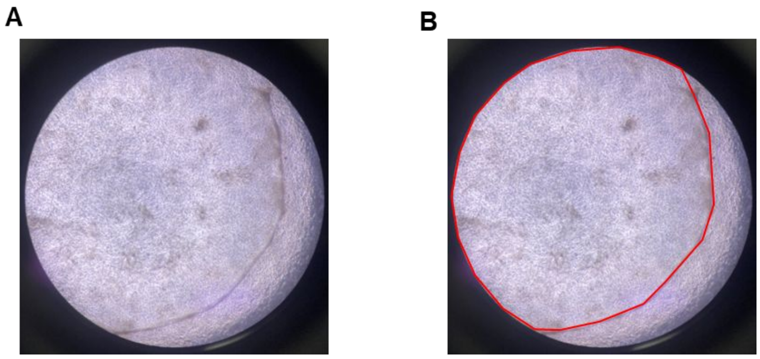

Figure S2. (A) Contraction of PIC-gel. (B) The red line represents the selection of the area used to calculate the contraction following the equation: Contraction = $(A_0 - A_t)/A_0 \times 100\%$, where A_0 is the area of the well, and A_t is the area of the contracted hydrogel.

Table S1. Characteristic properties of the PIC-hydrogels

**Hydrogel**	**Polymer**	***c*** **(g L^{-1})**	M_w **(kg mol^{-1})**	**$\langle d \rangle$ (nm)**	E_{eff} **(Pa)**	G' **(Pa)**	σ_c **(Pa)**
**PIC-0.2%**	PIC-RGD	2.0	309	10	258	161	19
**PIC-0.4%**	PIC-RGD	4.0	309	10	805	657	38
**PIC-0.6%**	PIC-RGD	6.0	309	10	1253	1160	N/A

c = polymer concentration, M_w = molecular weight of PIC-polymer $\langle d \rangle$ = average distance between RGD ligands in a polymer chain, E_{eff} = Effective modulus of hydrogel surface on day 0, G' = storage shear modulus at 37 ˚C, σ_c = critical stress

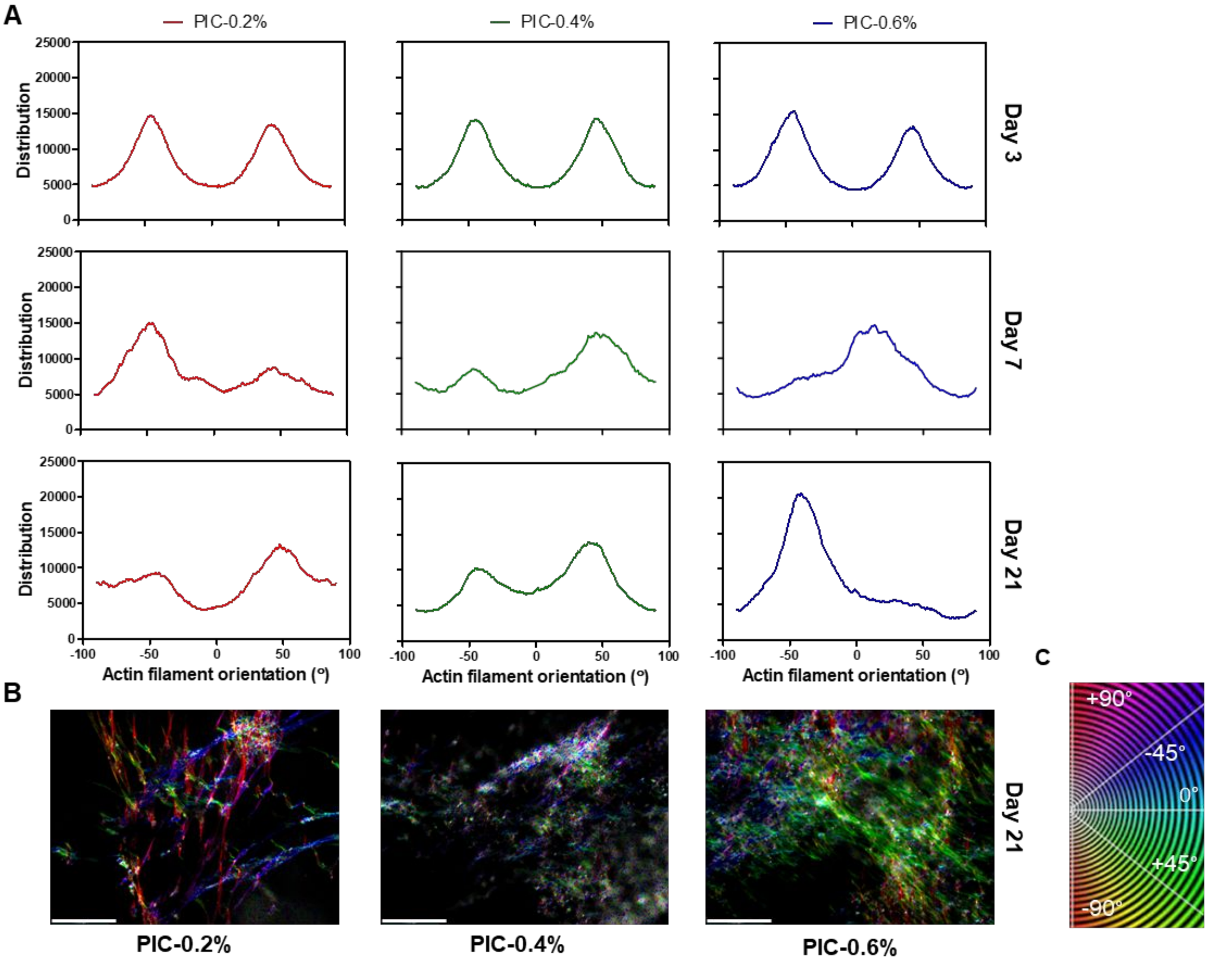

Figure S3. (A) The single distribution peak for vaginal fibroblast on PIC-0.6% after 7 days suggests the development of one dominant actin filament orientation in contrast to more distributed filament orientations on the other hydrogels. (B) A color survey of the morphology images on day 21 shows a variety of colors on PIC-0.2% and PIC-0.4% and a dominant (green) color on PIC-0.6% that correspond to a certain orientation (scale bars = 200 μm). (C) The relation between the colors and orientations.
